# Hybrid magnonics in hybrid perovskite antiferromagnets

**DOI:** 10.1038/s41467-023-37505-w

**Published:** 2023-04-01

**Authors:** Andrew H. Comstock, Chung-Tao Chou, Zhiyu Wang, Tonghui Wang, Ruyi Song, Joseph Sklenar, Aram Amassian, Wei Zhang, Haipeng Lu, Luqiao Liu, Matthew C. Beard, Dali Sun

**Affiliations:** 1 Department of Physics, North Carolina State University, Raleigh, NC 27695 USA; 2 Organic and Carbon Electronics Laboratory (ORACEL), North Carolina State University, Raleigh, NC 27695 USA; 3 Department of Electrical Engineering and Computer Science, Massachusetts Institute of Technology, Cambridge, MA 02139 USA; 4 Department of Chemistry and Energy Institute, The Hong Kong University of Science and Technology, Kowloon, 999077 Hong Kong (SAR) China; 5 Department of Materials Science and Engineering, North Carolina State University, Raleigh, NC 27695 USA; 6 Department of Mechanical Engineering and Material Science, Duke University, Durham, NC 27708 USA; 7 Department of Physics and Astronomy, Wayne State University, Detroit, MI 48202 USA; 8 Department of Physics and Astronomy, University of North Carolina at Chapel Hill, Chapel Hill, NC 27599 USA; 9 Chemistry and Nanoscience Center, National Renewable Energy Laboratory, Golden, CO 80401 USA

**Keywords:** Spintronics, Magnetic properties and materials, Quantum information, Organic-inorganic nanostructures

## Abstract

Hybrid magnonic systems are a newcomer for pursuing coherent information processing owing to their rich quantum engineering functionalities. One prototypical example is hybrid magnonics in antiferromagnets with an easy-plane anisotropy that resembles a quantum-mechanically mixed two-level spin system through the coupling of acoustic and optical magnons. Generally, the coupling between these orthogonal modes is forbidden due to their opposite parity. Here we show that the Dzyaloshinskii–Moriya-Interaction (DMI), a chiral antisymmetric interaction that occurs in magnetic systems with low symmetry, can lift this restriction. We report that layered hybrid perovskite antiferromagnets with an interlayer DMI can lead to a strong intrinsic magnon-magnon coupling strength up to 0.24 GHz, which is four times greater than the dissipation rates of the acoustic/optical modes. Our work shows that the DMI in these hybrid antiferromagnets holds promise for leveraging magnon-magnon coupling by harnessing symmetry breaking in a highly tunable, solution-processable layered magnetic platform.

## Abstract (web summary)

Recently there has been interest in exploring the coupling between magnons for use in information processing, however, this is hampered by the fact that such coupling is forbidden due to the different parity of the acoustic and optical magnons. Here, Comstock et al show that the interlayer Dzyaloshinskii–Moriya-Interaction in a layered hybrid antiferromagnet can allow for strong coupling between the acoustic and optical magnons, offering a pathway for magnon coherent information processing.

## Introduction

**Fig. 1:**
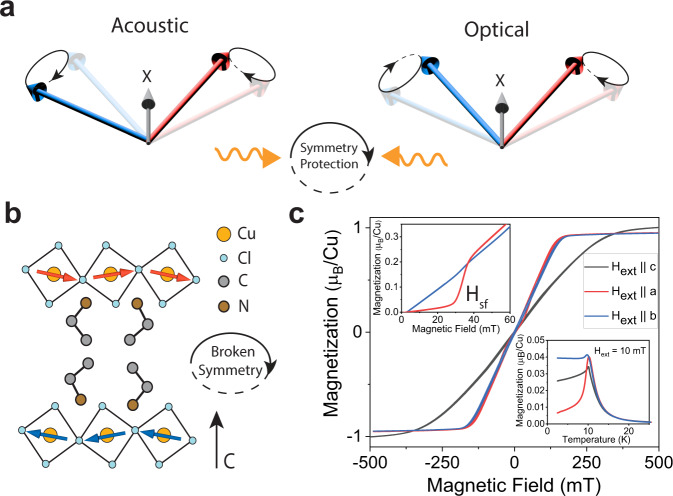
Magnetic properties of a hybrid perovskite antiferromagnet. **a** Schematic illustration of the acoustic and optical modes in a two-sublattice easy-plane antiferromagnet that are protected from interacting by parity. **b** A sketch of the inherently low symmetry of the Cu-EA structure due to octahedral tilting and the spin ordering of the layered antiferromagnet and its sublattice structure. **c** Magnetic properties of Cu-EA at *T* = 2.5 K. M(H) loops are obtained along all three principal axes of the single crystal up to saturation region. The upper inset shows the spin-flop transition at low field at $\left|{{{{{{\bf{H}}}}}}}_{{{{{{\bf{ext}}}}}}}\right |=30\,{{{{{\rm{mT}}}}}}$. M vs. T is included in the lower inset to show the onset of magnetic ordering at the transition temperature, *T* ~ 10 K.

The Dzyaloshinskii–Moriya interaction (DMI) arises in magnetic materials as a consequence of broken inversion symmetry and spin-orbit coupling (SOC)^1–3^. As a unique rotational sense of spins, the DMI is the essential phenomenon responsible for non-collinear antiferromagnets (AFMs)^4^ and the formation of chiral spin textures, such as spin spirals^5^, skyrmions^6^, and homochiral Néel-type domain walls^7^ with outstanding potential to store, transport, and process magnetic information^8,9^. Beyond static magnetization, DMI phenomena has recently moved into the forefront of interest particularly in AFMs due to its ability to also govern and control magnetic dynamics, i.e., collective spin excitations (magnons)^10,11^. At zero field, AFMs with easy-plane type of magnetic anisotropy exhibit high-frequency optical magnons with even parity (under a twofold rotation) and low-frequency acoustic magnons with odd parity whose frequencies can be tuned by an external field^12^ (Fig. 1a). The DMI is known to play a critical role of modifying the ellipticity of the precessional motion of the sublattice magnetizations of acoustic magnons, resulting in a dramatic enhancement of the spin injection efficiency via spin-pumping^13^. However, the correlation between the DMI and the high-frequency optical magnons remains elusive due to uncommon sub-terahertz microwave frequencies required for conventional AFMs^14–16^. Identifying new magnetic materials with the exceptional DMI in a more accessible frequency range empowers investigations of its microscopic origin and enables topological objects with non-trivial static and dynamic spin textures that stimulate fast, low-powered energy-efficient hybrid magnonic applications for coherent information processing^17,18^.

One such material class in this regard is the two-dimensional layered Cu-based magnetic hybrid organic–inorganic perovskites (HOIPs)^19–21^. Cu-based HOIPs consists of corner-sharing halogen, X = (Cl, Br) octahedra with the Cu atom situated at the center^22^. The strongly connected but tilted inorganic anion layers of corner-sharing CuX_4_^2-^ octahedra allow for a superexchange interaction via the Cu-X-Cu pathway (Figs. 1b, c), exhibiting magnetic ordering and multiferroic properties^23,24^. The naturally reduced symmetry in layered HOIPs^25^ provides an ideal platform for a three-site exchange with broken parity of the lattice that manifest the DMI^1^. Remarkably, the versatility of the organic cations offers a wide range of possibilities for synthetic control, e.g., interlayer spacer distance and inorganic-induced symmetry breaking distortions where a modulation of the weak interlayer AFM interaction or DMI can be achieved utilizing different sizes of organic cations. Incorporation of chiral organic cations will further break the symmetry and enable the chiral-induced-spin-selectivity (CISS) effect for promising spintronic applications^26–28^. The weak, synthetically tunable AFM interaction^29^ lowers both the frequencies of acoustic and optical magnons down to the easily accessible gigahertz frequency range^30^, making the study of rich synergistic effects between AFM magnons and the DMI feasible, opening up a broad field of physical phenomena.

Here we demonstrate that the presence of DMI, which acts as a symmetry breaking term in the Hamiltonian, in a layered AFM hybrid Cu-halide single crystal, (CH_3_CH_2_NH_3_)_2_CuCl_4_ (Cu-EA), enables hybridization between otherwise separate optical and acoustic modes and mimics a two-state character near the crossing point. In principle, due to symmetry protection, the two orthogonal magnon modes cannot interact and thus are present independently, allowing for a degeneracy to occur^31^. However, by breaking the symmetry under a twofold rotation induced by the DMI, these two modes couple, lifting the degeneracy and forming new hybrid states. This magnon–magnon coupling is identified by its typical signature which is an anticrossing gap occurring at the acoustic-optical crossing point. This coupling between the optical and acoustic magnons allows for the coherent transfer of spin information between these two quasi-particles^32–34^.

## Results

Magnetic properties of Cu-EA, a well-known a-type antiferromagnet^29^ are obtained by characterizing the magnetic hysteresis loops at *T* = 2.5 K as shown in Fig. 1c. By applying the magnetic field along the *a*- and *b*-axis, the M-H curve roughly saturates near $2{H}_{E} \sim 150{{{{{\rm{mT}}}}}}$ where ${H}_{E}$ is the interlayer antiferromagnetic exchange field. Along the *c*-axis, the saturation field is about 300 mT. The difference in the saturation field between the *c*-axis and the a-b plane represents planar magnetic anisotropy, probably caused by the shape anisotropy of the isolated magnetic CuCl_4_^2−^ layers separated by nonmagnetic (CH_3_CH_2_NH_3_^+^)_2_ spacers. At low external field (${{{{{\rm{|}}}}}}{{{{{{\bf{H}}}}}}}_{{{{{{\bf{ext}}}}}}}{{{{{\rm{|}}}}}}$~ 30 mT, top inset of Fig. 1c), a clear spin-flop transition at 30 mT is observed when the magnetic field is applied along the *a*-axis, suggesting a weak uniaxial magnetic anisotropy along this direction. Since the spin-flop transition occurs only at relatively low field, Cu-EA can be treated as an easy-plane antiferromagnet where the *c*-axis is the hard axis^29,35^. At high field above the spin-flop transition (${{{{{\rm{|}}}}}}{{{{{{\bf{H}}}}}}}_{{{{{{\bf{ext}}}}}}}{{{{{\rm{|}}}}}}$ > 30 mT), the M-H curves are nearly identical along both the *a*- and *b*-axis. From the M-H curves, it has been calculated^29,36^ that the planar magnetic anisotropy field (~215 mT) is much larger than the weak uniaxial anisotropy field (~7 mT) which further confirms the dominant easy-plane behavior (see S.I section III). In the bottom inset of Fig. 1c, the magnetization as a function of temperature is recorded, showing the onset of magnetic ordering below *T* = 10 K.

Presence of the DMI in Cu-EA has been extensively characterized^36,37^. The Hamiltonian for the DMI can be written as ${{{{{{\mathcal{H}}}}}}}_{{{{{{{\mathrm{DMI}}}}}}}}=-{{{{{\bf{D}}}}}}\cdot ({\hat{{{{{{\bf{m}}}}}}}}_{{{{{{\bf{A}}}}}}}{{{{\times }}}}{\hat{{{{{{\bf{m}}}}}}}}_{{{{{{\bf{B}}}}}}})$ where ${\hat{{{{{{\bf{m}}}}}}}}_{{{{{{\bf{A}}}}}}}$ and ${\hat{{{{{{\bf{m}}}}}}}}_{{{{{{\bf{B}}}}}}}$ are the magnetization unit vectors of each layer, and ${{{{{\bf{D}}}}}}$ is the DMI vector, which prefers orthogonal alignment of each sublattice magnetization, or spin canting^1^. Because the space group is Pbca^38^, the DMI vector is constrained to lie along the a-axis that is parallel to the easy axis (S.I. section I). Therefore, at zero field no spin canting will occur, resulting in the absence of weak ferromagnetism (S.I. section II). Only above the spin-flop transition the weak ferromagnetism manifests by which the strength of the DMI vector ($|{\mu }_{0}{{{{{\bf{D}}}}}}|$ ~ 11 mT) was determined using torque magnetometry and by studying the spin-flop transition^36,37^.

**Fig. 2:**
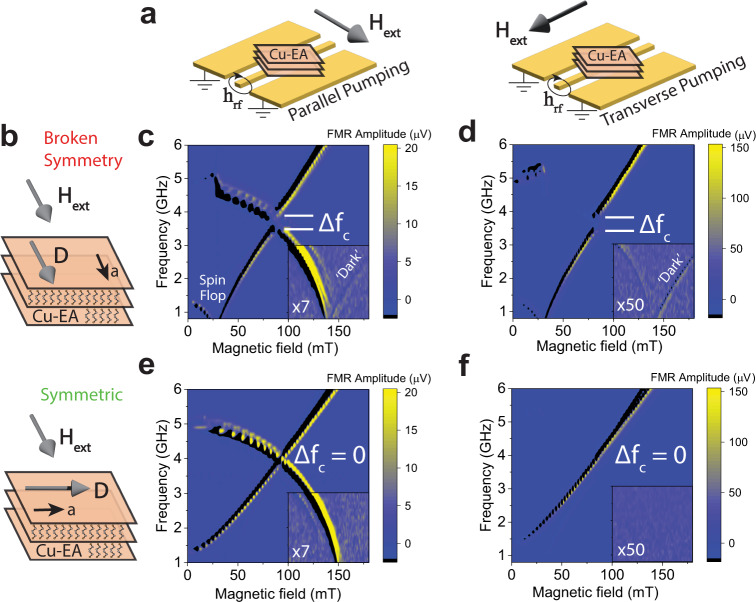
Antiferromagnetic resonance in Cu-EA single crystals with a strong magnon–magnon coupling induced by the DMI. **a** Schematic illustrations of the parallel and transverse pumping geometries for selectively exciting the optical and acoustic modes when the external magnetic field is applied parallel and perpendicular to the microwave RF field, ${{{{{{\bf{h}}}}}}}_{{{{{{\bf{rf}}}}}}}$. **b** Schematic illustrations of the broken symmetry (${{{{{{\bf{H}}}}}}}_{{{{{{\bf{ext}}}}}}}\parallel {{{{{\bf{D}}}}}}$) and symmetric (${{{{{{\bf{H}}}}}}}_{{{{{{\bf{ext}}}}}}}\perp {{{{{\bf{D}}}}}}$) pumping configurations when the external magnetic field is applied parallel and perpendicular to the DMI vector of the Cu-EA single crystal. **c**–**f** antiferromagnetic resonance spectra collected under the four possible experimental geometries as described in **a** and **b**. Insets in **c**–**f** show the resonance spectra under an adjusted color scale to elucidate the presence of dark modes at high fields.

Frequency-field dependence of the optical and acoustic modes in Cu-EA is studied using magnetic resonance (Fig. 2a). Due to the opposite symmetry of the optical and acoustic modes, two different microwave pumping geometries are applied allowing for their selective excitation. In the transverse pumping geometry (${H}_{\perp }$), the microwave magnetic field, **h**_**rf**_ should only be able to excite the acoustic mode. In the parallel pumping geometry (${H}_{\parallel }$), the in-plane component of **h**_**rf**_ has even parity, exciting the optical mode, while the out-of-plane component of **h**_**rf**_ can excite the acoustic mode, thus both modes can be observed^30^. To evaluate the effects of DMI on the magnon–magnon coupling, magnetic resonance is recorded with the field either parallel or perpendicular to ${{{{{\bf{D}}}}}}$, as shown in Fig. 2b. Figure 2c–f shows the measured frequency-field dependence of the antiferromagnetic resonance (AFMR) of Cu-EA in each of the experimental geometries described in Fig. 2a, b. In Fig. 2c, d, a dip in the acoustic mode frequency, as well as a discontinuity in the optical mode frequency is observed at 30 mT, which is an indication of the spin-flop transition^39^, consistent with the behavior of the MH loops for the magnetic field applied along the *a*-axis and parallel to the DMI vector. Additionally, the optical mode frequency for all four configurations reaches zero at 150 mT, consistent with the saturation field obtained from magnetometry and the assumption to treat Cu-EA as an easy-plane antiferromagnet. The ambient temperature during these measurements is $T=2.5{{{{{\rm{K}}}}}}$, far below the transition temperature of 10 K. The temperature dependence of these modes approaching the magnetic transition is shown in S.I. Fig. S10.

Both the acoustic and optical modes of the AFM resonance are clearly resolved in the gigahertz frequency range due to the weak AFM interlayer coupling, similar to that of layered AFM CrCl_3_^30^. Two features stand out: (i) A nonzero anticrossing gap, ${\triangle f}_{c}={{\min }}({f}_{\uparrow }-{f}_{\downarrow }) \sim 0.48\,{{{{{\rm{GHz}}}}}},$ forms between the two magnon modes, where ${f}_{\uparrow }$ and ${f}_{\downarrow }$ are the resonant frequencies of the upper and lower branches, respectively. A large $\Delta {f}_{c}$ is observed even though there is no oblique magnetic field applied^31^ but only when ${{{{{{\bf{H}}}}}}}_{{{{{{\bf{ext}}}}}}}\parallel {{{{{\bf{D}}}}}}$, indicating that the two modes are directly coupled through the DMI; (ii) The optical mode, which decreases in frequency with increasing field below ${{{{{{\bf{|H}}}}}}}_{{{{{{\bf{ext}}}}}}}{{{{{\rm{|}}}}}}=2{H}_{E}$ continues above $2{H}_{E}$ which is known to be a ‘dark’ mode^40^ and has even parity. Above $2{H}_{E}$, this ‘dark’ optical mode increases frequency with increasing field. This mode is only observed when ${{{{{{\bf{H}}}}}}}_{{{{{{\bf{ext}}}}}}}\parallel {{{{{\bf{D}}}}}}$ as shown in the bottom right of Fig. 2c and d, where the color scale has been adjusted to highlight this weak response. It is noteworthy that the dark mode increases intensity in the transverse pumping geometry, indicating that it can interact with the odd parity microwave field. Both the nonzero anticrossing gap ${\triangle f}_{c}$ at 100 mT and the dark mode above $2{H}_{E}$ are only observed when ${{{{{{\bf{H}}}}}}}_{{{{{{\bf{ext}}}}}}}\parallel {{{{{\bf{D}}}}}}$, confirming that the DMI plays the central role in breaking the symmetry that leads to these effects.

**Fig. 3:**
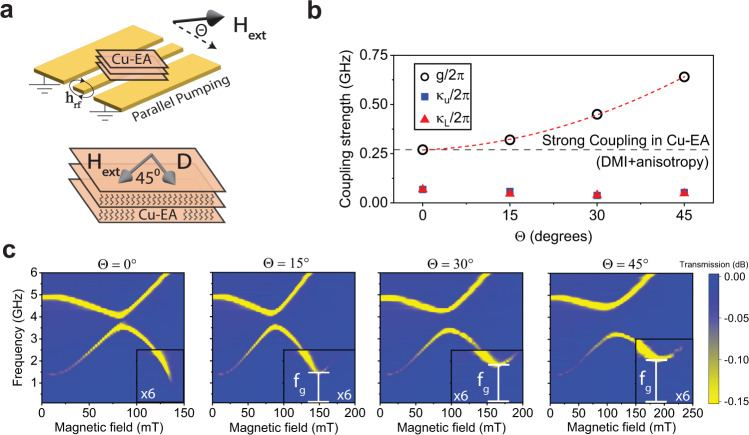
Enhanced magnon–magnon coupling strength. **a** Enhancement of the anticrossing gap by by rotating the external magnetic field by an oblique angle Θ along the out-of-plane direction at $45^\circ$ to the in-plane principal *a*-axis as indicated. **b** $\Theta$ dependence of the coupling strength ${g}_{c}$ and the dissipation rates for the upper and lower magnonic branches, ${\kappa }_{U}$ and ${\kappa }_{L}$ derived from the frequency-field dependence of resonance spe**c**tra presented in (**c**). The dotted red line is the fitted curve for the coupling strength following a quadratic behavior. The insets in (**c**) highlight the resonance spectra under an adjusted color scale, showing the increased DMI-induced nonzero gap, ${f}_{g}$ of the ‘dark’ mode as $\Theta$ increases.

To further analyze the strength of the magnon–magnon coupling, angular-dependent measurements are performed as described in Fig. 3a. Considering that the in-plane uniaxial magnetic anisotropy may also introduce symmetry breaking^41^, here we aim to increase the coupling further by applying the magnetic field at $45^\circ$ to the *a*-axis (S.I Fig. S7). Therefore, the resulting coupling arises from a combination of DMI and magnetic anisotropy. The magnon–magnon coupling strength, ${g}_{c}$ is determined as the half of the gap size between the optical and acoustic mode frequencies at their crossing point^42–44^ (i.e., $\frac{{g}_{c}}{2{{{{{\rm{\pi }}}}}}}=\left|{\triangle f}_{c}/2\right|$). We found that the coupling strength in Cu-EA reaches up to 0.27 GHz at *T* = 2.5 K, while the dissipation rates for the upper (${\kappa }_{U}/2\pi$) and lower (${\kappa }_{L}/2\pi$) branches given by the half widths at half maximum of the vertical cuts in the spectrum at the crossing point are roughly four times smaller than ${g}_{c}/2\pi$, satisfying the condition for a strong coupling: *g*_*c*_ > *κ*_*u*_ and *g*_*c*_ > *κ*_*l*_^43,45^. This coupling strength of 0.27 GHz due to the combination of DMI and magnetic anisotropy is only slightly larger than that due to the DMI alone, which is 0.24 GHz. When the external magnetic field is rotated to a larger out-of-plane (OOP) angle $\Theta$ (Fig. 3a), a symmetry breaking of the twofold rotation is further introduced in the sublattice magnetizations, enhancing the strength of the magnon–magnon coupling^46^. The magnitude of ${g}_{c}$ increases whereas ${\kappa }_{u}$ and ${\kappa }_{l}$ are largely independent of Θ, leading to a more pronounced coupling increasing from 0.27 GHz (Θ = 0°) to 0.64 GHz (Θ = 45°), consistent with previous observations indicating tunable magnon–magnon coupling^47^. We also verify that the anticrossing gap observed at Θ = 0° was not due to unintentional sample misalignment. A large cooperativity $C={g}^{2}/({\kappa }_{u}{\kappa }_{l})$ is obtained and continuously increased from ~15 at Θ = 0° to ~160 at Θ = 45°, suggesting the coupling extends into the ultrastrong coupling regime which may be useful for simulating exotic quantum magnonics phenomena.

Remarkably, we notice a unique observation that the ‘dark’ optical mode does not reach zero frequency at |**H**_**ext**_|=2*H*_*E*_. An anomalous gap is formed (denoted by ${f}_{g}$) although this gap is not related to the magnon coupling between the acoustic and optical modes. This gap size also increases with increasing Θ and reaches 2 GHz at Θ = 45°. As shown in S.I. section III.3, such a gap should not exist if the magnetic moments in adjacent sublattices are parallel at ${{{{{\rm{|}}}}}}{{{{{{\bf{H}}}}}}}_{{{{{{\bf{ext}}}}}}}{{{{{\rm{|}}}}}} > 2{H}_{E}$ and can be attributed to the residual spin canting that arises from the DMI. This residual spin canting at ${{{{{\rm{|}}}}}}{{{{{{\bf{H}}}}}}}_{{{{{{\bf{ext}}}}}}}{{{{{\rm{|}}}}}} > 2{H}_{E}$ is another typical signature of the DMI in Cu-EA which prevents a collinear alignment of the sublattice magnetizations, causing them to cant slightly due to their preferred orthogonal alignment (see discussion in S.I. section II).

## Discussion

All the three unique features observed in the AFM resonance of Cu-EA single crystal, i.e., the formation of strong magnon–magnon coupling at Θ = 0°, the presence of even-parity ‘dark’ optical modes under the transverse excitation condition, and the anomalous gap at $\left|{{{{{{\bf{H}}}}}}}_{{{{{{\bf{ext}}}}}}}\right |=2{H}_{E}$ can be attributed to the DMI in the Cu-EA crystal (S.I. section V). For the key observation about the strong magnon–magnon coupling and nonzero anticrossing gap at Θ = 0°, we performed analytical calculations by employing a set of coupled Landau-Lifshitz^48^ (LL) equations with a two-sublattice model and the DMI term. The Hamiltonian of the DMI term is written as ${{{{{{\mathcal{H}}}}}}}_{{{{{{{\mathrm{DMI}}}}}}}}=-{{{{{\bf{D}}}}}}\cdot \left({\hat{{{{{{\bf{m}}}}}}}}_{{{{{{\bf{A}}}}}}}\times {\hat{{{{{{\bf{m}}}}}}}}_{{{{{{\bf{B}}}}}}}\right)$ $={\hat{{{{{{\bf{m}}}}}}}}_{{{{{{\bf{A}}}}}}}\cdot ({D}_{x}\left(\hat{{{{{{\bf{x}}}}}}}\times {\hat{{{{{{\bf{m}}}}}}}}_{{{{{{\bf{B}}}}}}}\right))+{\hat{{{{{{\bf{m}}}}}}}}_{{{{{{\bf{B}}}}}}}\cdot ({-D}_{x}\left(\hat{{{{{{\bf{x}}}}}}}\times {\hat{{{{{{\bf{m}}}}}}}}_{{{{{{\bf{A}}}}}}}\right))$, where ${D}_{x,y,z}$ are the components of the DMI vector that defines the direction of the inversion asymmetric exchange field, ${\hat{m}}_{A(B)}$ is the sublattice magnetic moment located on adjacent atomic sites *A*(*B)*. The direction of **D** is determined to be along the a-axis which for mathematical purposes here we define it as the x-direction. When ${{{{{{\bf{H}}}}}}}_{{{{{{\bf{ext}}}}}}}\parallel {{{{{\bf{D}}}}}}$, each sublattice experiences an effective field that is antisymmetric under twofold rotation, allowing interactions between the two modes with opposite parity. By including this effective field into the coupled LL equations, we obtain:

$$\frac{d{\hat{{{{{{\bf{m}}}}}}}}_{{{{{{\bf{A}}}}}}}}{{dt}}=-{\mu }_{0}\gamma {\hat{{{{{{\bf{m}}}}}}}}_{{{{{{\bf{A}}}}}}}\times \left({{{{{{\bf{H}}}}}}}_{{{{{{\bf{ext}}}}}}}-{H}_{E}{\hat{{{{{{\bf{m}}}}}}}}_{{{{{{\bf{B}}}}}}}-{M}_{s}\left({\hat{{{{{{\bf{m}}}}}}}}_{{{{{{\bf{A}}}}}}}\cdot \hat{{{{{{\bf{z}}}}}}}\right)\hat{{{{{{\bf{z}}}}}}}+{D}_{x}\left(\hat{{{{{{\bf{x}}}}}}}\times {\hat{{{{{{\bf{m}}}}}}}}_{{{{{{\bf{B}}}}}}}\right)\right)+{{{{{{\boldsymbol{\tau }}}}}}}_{{{{{{\bf{A}}}}}}},$$

1 $$\frac{d{\hat{{{{{{\bf{m}}}}}}}}_{{{{{{\bf{B}}}}}}}}{{dt}}=-{\mu }_{0}\gamma {\hat{{{{{{\bf{m}}}}}}}}_{{{{{{\bf{B}}}}}}}\times \left({{{{{{\bf{H}}}}}}}_{{{{{{\bf{ext}}}}}}}-{H}_{E}{\hat{{{{{{\bf{m}}}}}}}}_{{{{{{\bf{A}}}}}}}-{M}_{s}\left({\hat{{{{{{\bf{m}}}}}}}}_{{{{{{\bf{B}}}}}}}\cdot \hat{{{{{{\bf{z}}}}}}}\right)\hat{{{{{{\bf{z}}}}}}}-{D}_{x}\left(\hat{{{{{{\bf{x}}}}}}}\times {\hat{{{{{{\bf{m}}}}}}}}_{{{{{{\bf{A}}}}}}}\right)\right)+{{{{{{\boldsymbol{\tau }}}}}}}_{{{{{{\bf{B}}}}}}},$$

where γ is the gyromagnetic ratio, ${M}_{s}$ is the saturation magnetization of the sublattice, and ${{{{{{\boldsymbol{\tau }}}}}}}_{{{{{{\bf{A}}}}}}}$ and ${{{{{{\boldsymbol{\tau }}}}}}}_{{{{{{\bf{B}}}}}}}$ are the torques induced by the microwave rf field. The terms in parenthesis of Eq. (1) are the effective fields that each sublattice experiences due to the applied external magnetic field; exchange field; anisotropy term; and the DMI. We show (S.I. section III**)** that these equations can be re-written in the basis ${{{{{\boldsymbol{\delta }}}}}}{{{{{{\bf{m}}}}}}}_{{{{{{\boldsymbol{+}}}}}}}$ (optical) and ${{{{{\boldsymbol{\delta }}}}}}{{{{{{\bf{m}}}}}}}_{{{{{{\boldsymbol{-}}}}}}}$ (acoustic) and in matrix form: $${\omega }^{2}\begin{array}{c}{{{{{\boldsymbol{\delta }}}}}}{{{{{{\bf{m}}}}}}}_{{{{{{\boldsymbol{+}}}}}}}\\ {{{{{\boldsymbol{\delta }}}}}}{{{{{{\bf{m}}}}}}}_{{{{{{\boldsymbol{-}}}}}}}\end{array}=\left[\begin{array}{cc}{\omega }_{O}^{2} & \Delta \\ -\Delta & {\omega }_{A}^{2}\end{array}\right]\begin{array}{c}{{{{{\boldsymbol{\delta }}}}}}{{{{{{\bf{m}}}}}}}_{{{{{{\boldsymbol{+}}}}}}}\\ {{{{{\boldsymbol{\delta }}}}}}{{{{{{\bf{m}}}}}}}_{{{{{{\boldsymbol{-}}}}}}}\end{array}$$. If ${D}_{x}$ is nonzero, an off-diagonal term $\Delta$ appears which represents the strength of the DMI coupling. Here ${\omega }_{O}$ and ${\omega }_{A}$ are the bare optical and acoustic mode frequencies. We found that the experimentally measured coupling strength leads to a ${{\mu }_{0}D}_{x}$ value of 11 mT, consistent with micromagnetic simulations and previously reported experimental values^36^(S.I. section VI). This mechanism for the magnon–magnon coupling can mediate the conversion of a magnon of one parity to that of the alternate parity, resulting in the coherent transfer of information between these two magnon modes. Since the bare optical and acoustic modes are no longer eigenstates of the Hamiltonian, time dependence of their mutual conversion may exhibit Rabi-like oscillations^49^. The new eigenstates near the crossing point, thereby, take the form of a linear combination between the two bare modes and represents a newly formed two-state character.

The DMI field also accounts for the presence of optical modes in the transverse pumping configuration and the ‘dark’ optical mode at high fields. The antisymmetric nature of the DMI manifests as an effective microwave field **h**_**rf, DMI**_ that has opposite signs as experienced by the different spin sublattices. The application of a spatially uniform microwave field naturally exerts a torque on each sublattice which is in the same direction for the two sublattices and drives their in-phase motion. However, when the DMI manifests, this deflection will result in the effective **h**_**rf, DMI**_ in the alternate sublattice which points towards ${D}_{x}(\hat{{{{{{\bf{x}}}}}}}\times {{{{{\boldsymbol{\delta }}}}}}{\hat{{{{{{\bf{m}}}}}}}}_{{{{{{\bf{B}}}}}}})$for sublattice A and${-D}_{x}(\hat{{{{{{\bf{x}}}}}}}\times {{{{{\boldsymbol{\delta }}}}}}{\hat{{{{{{\bf{m}}}}}}}}_{{{{{{\bf{A}}}}}}})$ for sublattice B. This effective field **h**_**rf, DMI**_ reverses polarity at the same microwave frequency, and acts on opposite directions in each sublattice (S.I. Fig. S8). Due to this antisymmetric nature of the DMI, the torques applied to the sublattices allow for the dark mode to be observed. Even though **h**_**rf, DMI**_$\perp {{{{{{\bf{H}}}}}}}_{{{{{{\bf{ext}}}}}}}$, its sublattice-dependent direction causes it to be even under twofold rotation and allows it to transfer energy to the optical mode, as also observed in micromagnetic simulations (S.I. section VI).

Our results demonstrate the possibilities of layered 2D HOIP materials for strong and tunable hybrid magnonics by utilizing their low-dimensional magnetic dynamics and chemical versatility. By varying the organic spacer, the low symmetry of layered hybrid structure opens exciting perspectives for studying topological chiral spin textures induced by the DMI along with its tunability, and enables a strongly hybridized magnon state to emerge, resembling the quantum-mechanical coupling phenomena for quantum information science. Our work expands the scope of realizing solution-processed antiferromagnetic phenomena in the electronics-accessible gigahertz frequency range, holding promises for research surrounding synthetic antiferromagnet engineering and materials synthesis in the field of hybrid magnonics.

## Methods

### Synthesis of EA_2_CuCl_4_ single crystals

Cu-EA ((CH_3_CH_2_NH_3_)_2_CuCl_4_) single crystal was grown by controlled temperature cooling method. 652.32 mg EACl and 537.8 mg CuCl_2_ was added into 2 mL dimethylformamide (DMF) and stirred overnight at 100 °C to get completely dissolved. Then the solution was transferred to the oil bath preheated at 100 °C and cooled with the rate of 1 °C/h to room temperature. The precipitated yellow-brown color platelet single crystals were collected by vacuum filtration and further dried in the vacuum oven at 60 °C for 24 h. The crystals used in Fig. 3 were obtained by the following method: Copper(II) chloride dihydrate (CuCl_2_·2H_2_O, ≥ 99.95%), Ethylamine solution (EA, 66.0-72.0% in H_2_O) and hydrochloric acid (HCl, 37%) were all purchased from Sigma–Aldrich. 0.341 g (2 mmol) of CuCl_2_·2H_2_O and 333 μL (4 mmol) EA were dissolved in 2 ml of 37% HCl solution by heating to 100 °C under constant magnetic stirring. Then the mixed solution was naturally cooled to room temperature. Finally, the yellow plate-like crystals EA_2_CuCl_4_ were obtained and isolated by vacuum filtration.

#### UV-VIS spectroscopy

The absorption spectra were obtained by optical diffuse reflectance mode in the UH5700 spectrophotometer with a 60 mm diameter integrating sphere. They were measured in the range of 200–1300 nm with 0.5 nm resolution at room temperature. BaSO_4_ was used as the 100% reflectance background and the crystals were ground into powder and adhered to the barium sulfate background by pressing for measurement.

#### Powder X-ray diffraction

The powder X-ray diffraction data were collected on the PANalytical X’Pert Pro powder-X-ray diffractometer with a Cu source.

#### SQUID measurements

A single crystal of Cu-EA was obtained and secured between two small pieces of Kapton (polyamide) tape. These single crystals are relatively flat disks with the *c*-axis oriented normal to the plane of the crystal. The *a*-axis was determined by angular-dependent measurements to find the direction of the spin-flop transition. A Magnetic Property Measurement System (MPMS, Quantum Design) was used to measure all magnetic susceptibility data. The sample was secured to a quartz rod using Kapton tape for in-plane (IP) measurements and to the opening of a plastic straw for measurements in which the magnetic field is applied out of plane (OOP). For temperature dependent susceptibility, the magnetic moment was measured in a relatively low magnetic field of 10 mT and under the vibrating sample magnetometer (VSM) mode after cooling in zero field.

#### Antiferromagnetic resonance measurements

For the antiferromagnetic resonance (AFMR) measurements, the same single crystal of Cu-EA used for SQUID magnetometry was secured with Kapton tape to the waveguide of the commercially available FMR probe designed to be compatible with the Physical Property Measurement System (PPMS, Quantum Design). To reduce data collection times, the FMR spectrometer that was supplied with the probe was not used to supply the microwave power, but was used as a current source for the Helmholtz coil. A Keysight EXG Analog Signal Generator N5173B was used as a microwave power source with an amplitude of −2 dBm and frequencies between 0.8 and 5.5 GHz in 0.1 GHz steps. After passing by the crystal of Cu-EA, the signal was intercepted by a Krytar microwave power detector model 201B and the corresponding voltage was routed to an EG&G 7260 DSP lock in amplifier. The reference signal for the current source driving the Helmholtz coil (490 Hz) was output to the lock in amplifier. For parallel pumping measurements the sample was removed and re-mounted to the section of the waveguide strip running perpendicular to the external magnetic field. For azimuthal angle Φ dependent measurements the sample was removed from the waveguide and manually rotated.

#### Angular-dependent AFMR measurements

A vector network analyzer (model HP 8720 C) was used to measure the microwave transmission (S21) for the angular-dependent FMR measurements. The Cu-EA sample was fixed to a coplanar waveguide with Kapton tape. The azimuthal angle was determined by angular-dependent measurements along the a-b plane. The out-of-plane tilt angle Θ was varied by rotating the waveguide manually without re-mounting the sample nor changing the in-plane angle Φ. The measurement was done in a cryostat at 2.5 K with the probing RF power set at −10 dBm. The coplanar waveguide provides both parallel and transverse pumping, as shown in Fig. 2a.

## Supplementary information

Supplementary Information

## Data Availability

The source data for the Supplementary Information are available from the corresponding authors upon reasonable request. Source data are provided with this paper.

## Source data

Source Data
