# Supplementary material for: Hybrid magnonics in hybrid perovskite antiferromagnets
Source: Nat Commun. 2023 Apr 1;14:1834. doi: 10.1038/s41467-023-37505-w (PMC10067936; doi:10.1038/s41467-023-37505-w)
Supplement: Supplementary file 1 — Supplementary Information [file 41467_2023_37505_MOESM1_ESM.pdf]

Supplementary Information

Hybrid Magnonics in Hybrid Perovskite Antiferromagnets

Andrew H. Comstock[1,2†], Chung-Tao Chou[3†], Zhiyu Wang[4], Tonghui Wang[2,5], Ruyi Song[6], Joseph Sklenar[7], Aram Amassian[2,5], Wei Zhang[8], Haipeng Lu[4,9]*, Luqiao Liu[3]*, Matthew C. Beard[9]*, Dali Sun[1,2]*

[1] Department of Physics, North Carolina State University, Raleigh, NC 27695 USA
[2] Organic and Carbon Electronics Laboratory (ORACEL), North Carolina State University, Raleigh, NC 27695 USA
[3] Department of Electrical Engineering and Computer Science, Massachusetts Institute of Technology, Cambridge, MA 02139, USA
[4] Department of Chemistry and Energy Institute, The Hong Kong University of Science and Technology, Kowloon 999077 Hong Kong (SAR), China
[5] Department of Materials Science and Engineering, North Carolina State University, Raleigh, NC 27695 USA
[6] Department of Mechanical Engineering and Material Science, Duke University, Durham, NC 27708 USA
[7] Department of Physics and Astronomy, Wayne State University, Detroit, MI 48202, USA
[8] Department of Physics and Astronomy, University of North Carolina at Chapel Hill, NC 27599, USA
[9] Chemistry and Nanoscience Center, National Renewable Energy Laboratory, Golden, CO 80401, USA

Correspondence to: haipenglu@ust.hk, luqiao@mit.edu, matt.beard@nrel.gov, dsun4@ncsu.edu.

Contents:

Supplementary Text
I. DMI and determination of orientation of D
II. Spin configuration of Cu-EA under an applied field
III. Analytical LLG model with the DMI
IV. Possible additional sources for the observed anticrossing gap
V. Observation of magnon dark modes
VI. Numeric simulation
Figs. S1 to S14

I. DMI and determination of orientation of D

The Dzyaloshinskii-Moriya interaction is subject to certain symmetry constraints based on its antisymmetric nature. Moriya has quantified these constraints in 5 simple rules, of which we will only apply three[1]. The rules are summarized briefly, assuming sublattices joined by the line AB that is bisected by C:

a. When a center of inversion is located at C, $\mathbf{D} = 0$.
b. When a mirror plane perpendicular to AB passes through C, $\mathbf{D} \perp AB$.
c. When a twofold rotation axis perpendicular to AB passes through C, $\mathbf{D} \perp twofold\ axis$.

Cu-EA crystallizes in the Pbca space group[2,3] with a face-centered orthorhombic structure, where the Cu atoms are located at the inversion centers. From the symmetries of the crystal structure, it is evident that there are no mirror planes or twofold rotations, but these are replaced with glide planes and screw axes. The same symmetry constraints imposed by Moriya still hold, since the added translation does not change the direction of **D**. Only two symmetry elements are relevant here, the glide plane and screw axis which are oriented at $\frac{1}{4}$ the height of the unit cell along the c-axis and bisect the distance between the sublattices. Additionally, there are no inversion centers located between the two sublattices, so the DMI is allowed to exist. If it does exist, it must be ⊥ to the b-axis due to the screw axis, and ⊥ to the c-axis due to the glide plane[4,5]. Therefore, the only choice for the orientation of **D** is along the a-axis.

II. Spin configuration of Cu-EA under an applied field

Previously, magnetic susceptibility studies on Cu-EA have shown weak uniaxial anisotropy along a preferred in-plane direction, the a-axis. For the rest of the supplementary material, we will use (x, y, z) instead of (a, b, c), in order to be consistent with the main text. At zero applied field, the magnetization of each sublattice lies along the x axis (easy axis), and the DMI has no effect on the magnetization orientation since it is parallel to each magnetization ($\mathbf{D} \times \hat{\mathbf{m}}_\mathbf{A} = 0$).

As the strength of the magnetic field along the x axis is increased below a certain value, the spin configuration remains unchanged. However, once the field strength reaches a certain value $H_{sf} \sim 30mT$ the spin flop transition will occur, and the magnetization of each sublattice will reorient along the y axis in a first order transition. At this point, the covert spin canting from the DMI becomes overt and the antisymmetric exchange becomes relevant. Here, the symmetry is broken under C_{2x}. As the magnetic field strength is further increased, the sublattice magnetizations begin to tilt toward the x direction with angle φ until they become fully aligned with the a-axis at $|\mathbf{H}_\mathbf{ext}| = 2H_E$.

When the magnetic field is applied along the y axis, no spin flop transition occurs. Instead, there is a canting of the magnetization towards the y axis with angle φ which occurs simultaneously with a tilting of one sublattice toward z and one toward –z. Here, the symmetry is preserved under C_{2y} (rotation is performed about the applied field, which is in the y-direction). At $|\mathbf{H}_\mathbf{ext}| = 2H_E$ the sublattice magnetizations are nearly aligned with the applied field except for some small tilting in the z-direction. As the field strength is increased, the magnetic moment

can be further increased even past the expected saturation field. These processes are described in **Figure S3**.

III. Analytical LLG model with the DMI

In order to derive the analytic model for the LLG equations in the main text, the assumption that the magnetization of each sublattice can be treated in the macrospin approximation is made. The free energy per magnetic moment, then, is written as:

$$F_m = \frac{F}{m_A} = -\mu_0 \mathbf{H}_{\mathbf{ext}} \cdot (\hat{\mathbf{m}}_{\mathbf{A}} + \hat{\mathbf{m}}_{\mathbf{B}}) + \mu_0 H_E (\hat{\mathbf{m}}_{\mathbf{A}} \cdot \hat{\mathbf{m}}_{\mathbf{B}}) - \mu_0 \mathbf{D} \cdot (\hat{\mathbf{m}}_{\mathbf{A}} \times \hat{\mathbf{m}}_{\mathbf{B}}) + \mu_0 M_s/2((\hat{\mathbf{m}}_{\mathbf{A}} \cdot \hat{\mathbf{c}})^2 + (\hat{\mathbf{m}}_{\mathbf{B}} \cdot \hat{\mathbf{c}})^2) - \mu_0 K_a/2((\hat{\mathbf{m}}_{\mathbf{A}} \cdot \hat{\mathbf{a}})^2 + (\hat{\mathbf{m}}_{\mathbf{B}} \cdot \hat{\mathbf{a}})^2)$$

where $\mathbf{H}_{\mathbf{ext}}$ is the external applied field, $\hat{\mathbf{m}}_{\mathbf{A}}$ and $\hat{\mathbf{m}}_{\mathbf{B}}$ are magnetization of each sublattice, H_E is the exchange field, $\mathbf{D}$ is the DMI vector, and M_s and K_a are parameters relating to the easy-plane and uniaxial anisotropy, respectively. Due to the fitting of the LLG models, as well as previously reported values[6] $\mu_0 M_s = 215mT$ and $\mu_0 K_a = 7mT$. Since we are interested in the physics at and above the crossing point (~100mT) we ignore the uniaxial anisotropy in our analytical equations. Moreover, the easy plane anisotropy is much stronger than the weak uniaxial anisotropy so that Cu-EA is treated as an easy-plane antiferromagnet.

To obtain equation 1 of the main text, the LLG equations are derived[7] by:

$$\frac{d\hat{\mathbf{m}}_{\mathbf{A,B}}}{dt} = -\gamma(\hat{\mathbf{m}}_{\mathbf{A,B}} \times \frac{dF_m}{d\hat{\mathbf{m}}_{\mathbf{A,B}}})$$

III.1. Analytical LLG model for the DMI- induced anticrossing gap

Here we assert that the DMI has an effect on the equilibrium sublattice magnetization direction, which acts similarly to an oblique magnetic field to tilt the sublattice magnetizations OOP. However, we show that it also can hybridize the optical and acoustic magnons due to its time-varying effect which is present even if the equilibrium magnetization orientation is symmetric. We will refer to these effects as the "passive" and "active" DMI, respectively. In order to analyze and separate the contributions from the active and passive DMI, we will solve the coupled LLG equations under two criteria; 1) the equilibrium magnetic moments are symmetric under twofold rotation and lie IP, but the DMI is present in the coupled LLG equations (active), and 2) the DMI-induced spin canting tilts the equilibrium magnetizations OOP but this is the only source of magnon-magnon coupling (passive). In these models, we will always set $\mathbf{H}_{\mathbf{ext}} \parallel \hat{\mathbf{x}}$ and change the direction of $\mathbf{D}$ between the x and y directions. Because the anisotropies along the xy plane are relatively small and are only relevant at low field ranges (<25 mT), we will ignore their effects for now in order to study the physics near the crossing point (~85 mT). At zero field, the equilibrium spins will align along the y axis and tilt toward the applied field direction with angle φ as the field strength is increased, as illustrated in **Figure S4.**

First, we will study the case in which $\mathbf{H_{ext}} \parallel \mathbf{D} \parallel \hat{\mathbf{x}}$. Assuming harmonic time dependence $\hat{\mathbf{m}}_{\mathbf{A,B}} = \hat{\mathbf{m}}^{\mathbf{eq}}_{\mathbf{A,B}} + \boldsymbol{\delta}\mathbf{m}_{\mathbf{A,B}}\exp(i\omega t)$ substituted into equation 1 of the main text and keeping linear order terms only, we obtain:

$$i\omega\boldsymbol{\delta}\mathbf{m_A} = \mu_0\gamma\hat{\mathbf{m}}^{\mathbf{eq}}_{\mathbf{A}} \times \left(H_{eq}\boldsymbol{\delta}\mathbf{m_A} + H_E\boldsymbol{\delta}\mathbf{m_B} + M_s(\boldsymbol{\delta}\mathbf{m_A}\cdot\hat{\mathbf{z}})\hat{\mathbf{z}} - D_x(\hat{\mathbf{x}} \times \boldsymbol{\delta}\mathbf{m_B})\right)$$
$$i\omega\boldsymbol{\delta}\mathbf{m_B} = \mu_0\gamma\hat{\mathbf{m}}^{\mathbf{eq}}_{\mathbf{B}} \times \left(H_{eq}\boldsymbol{\delta}\mathbf{m_B} + H_E\boldsymbol{\delta}\mathbf{m_A} + M_s(\boldsymbol{\delta}\mathbf{m_B}\cdot\hat{\mathbf{z}})\hat{\mathbf{z}} + D_x(\hat{\mathbf{x}} \times \boldsymbol{\delta}\mathbf{m_A})\right)$$

where $H_{eq}\hat{\mathbf{m}}^{\mathbf{eq}}_{\mathbf{A}} = \mathbf{H_{ext}} - H_E\hat{\mathbf{m}}^{\mathbf{eq}}_{\mathbf{B}} - D_x\left(\hat{\mathbf{x}} \times \hat{\mathbf{m}}^{\mathbf{eq}}_{\mathbf{B}}\right)$ is defined. We will assume equilibrium magnetic configurations such that $C_{2x}\hat{\mathbf{m}}^{\mathbf{eq}}_{\mathbf{B}} = \hat{\mathbf{m}}^{\mathbf{eq}}_{\mathbf{A}}$ which preserve rotation symmetry since the canting angle is assumed small because $D_x \ll H_E$. To obtain an equation for $\boldsymbol{\delta}\mathbf{m}_+$ we will act on the second equation with C_{2x} and add it to the first, and for $\boldsymbol{\delta}\mathbf{m}_-$ we will subtract them. Note that $C_{2x}D_x(\hat{\mathbf{x}} \times \boldsymbol{\delta}\mathbf{m_A}) = D_x(\hat{\mathbf{x}} \times C_{2x}\boldsymbol{\delta}\mathbf{m_A})$. We arrive at the following

$$i\omega\boldsymbol{\delta}\mathbf{m}_\pm = \mu_0\gamma\hat{\mathbf{m}}^{\mathbf{eq}}_{\mathbf{A}} \times (H_E\boldsymbol{\delta}\mathbf{m}_\pm \pm H_E C_{2x}\boldsymbol{\delta}\mathbf{m}_\pm + M_s(\boldsymbol{\delta}\mathbf{m}_\pm\cdot\hat{\mathbf{z}})\hat{\mathbf{z}}) \mp \mu_0\gamma D_x\left(\hat{\mathbf{m}}^{\mathbf{eq}}_{\mathbf{A}}\cdot\hat{\mathbf{x}}\right)C_{2x}\boldsymbol{\delta}\mathbf{m}_\mp$$
$$\pm \mu_0\gamma D_x\hat{\mathbf{x}}(\hat{\mathbf{m}}^{\mathbf{eq}}_{\mathbf{A}}\cdot C_{2x}\boldsymbol{\delta}\mathbf{m}_\mp)$$

Here it becomes obvious that these equations are not easily decoupled due to the D_x term. Writing them in matrix form with the basis $\boldsymbol{\delta}\mathbf{m}_\pm = \delta m_{\varphi\pm}\,\hat{\boldsymbol{\varphi}} + \delta m_{\theta\pm}\,\hat{\boldsymbol{\theta}}$ we obtain off diagonal matrix elements as shown below:

$$i\omega\begin{matrix}\boldsymbol{\delta}\mathbf{m}_+\\ \boldsymbol{\delta}\mathbf{m}_-\end{matrix} = \mu_0\gamma\begin{bmatrix} A & C \\ -C & B\end{bmatrix}\begin{matrix}\boldsymbol{\delta}\mathbf{m}_+\\ \boldsymbol{\delta}\mathbf{m}_-\end{matrix}$$

The elements of that matrix are as follows:

$$A = \begin{bmatrix} 0 & -M_s \\ 2H_E\cos^2\varphi & 0\end{bmatrix}$$

$$B = \begin{bmatrix} 0 & -2H_E - M_s \\ 2H_E\sin^2\varphi & 0\end{bmatrix}$$

$$C = D_x\begin{bmatrix} \cos\varphi\sin 2\varphi - \sin\varphi\cos 2\varphi & 0 \\ 0 & \sin\varphi\end{bmatrix}$$

where C is the off-diagonal element. Therefore, the problem is reduced to a simple eigenvalue equation where the eigenvalues ω can be determined by subtracting $i\omega 1_4$ from $\mu_0\gamma\begin{bmatrix} A & C \\ -C & B\end{bmatrix}$ and setting the determinant equal to zero. By taking the relationship between the external applied field and H_E as $\sin\varphi = \frac{|\mathbf{H_{ext}}|}{2H_E}$ we precisely reproduce the experimental results.

Above, we have completed the analytic LLG model for $\mathbf{D} \parallel \mathbf{H_{ext}}, \hat{\mathbf{x}}$ and now we are ready to consider $\mathbf{D} \parallel \hat{\mathbf{y}}, \mathbf{H_{ext}} \parallel \hat{\mathbf{x}}$. We will follow the same methodology as above, but now we note that $C_{2x}D_y(\hat{\mathbf{y}} \times \boldsymbol{\delta}\mathbf{m_A}) = -D_y(\hat{\mathbf{y}} \times C_{2x}\boldsymbol{\delta}\mathbf{m_A})$ allows us to diagonalize the matrix equation and separate the equations for $\boldsymbol{\delta}\mathbf{m}_+$ and $\boldsymbol{\delta}\mathbf{m}_-$ so no magnon-magnon coupling is predicted. We include the matrix elements for $\mathbf{D} \parallel \hat{\mathbf{y}}$ for completeness:

$$A = \begin{bmatrix} \mathrm{D_y}(\cos\varphi\cos 2\varphi + \sin\varphi\sin 2\varphi) & -M_s \\ 2H_E\cos^2\varphi & D_y\cos\varphi \end{bmatrix}$$

$$B = \begin{bmatrix} -\mathrm{D_y}(\cos\varphi\cos 2\varphi + \sin\varphi\sin 2\varphi) & -2H_E - M_s \\ 2H_E\sin^2\varphi & -D_y\cos\varphi \end{bmatrix}$$

$$C = \begin{bmatrix} 0 & 0 \\ 0 & 0 \end{bmatrix}$$

So, the matrix elements are zero on the off-diagonals, again predicting no magnon-magnon coupling, but are nonzero on the diagonals. Finally, to quantify the contribution of D_x for different orientations of the applied field, we plot the eigenvalues to replicate the experimental data. By setting $\mu_0 H_E = 75mT$, $\mu_0 M_s = 215mT$, $\gamma = 28\ GHz/T$ and $\mu_0 D_x = 11mT$ we can accurately reproduce the experimental data as shown in **Figure S5**.

Based on the literature, the DMI strength in Cu-EA has previously been determined to be ~11.9 mT[8], which is a very good fit to our experimental results. The calculated coupling strength $g/2\pi$ resulting from a DMI strength of 11 mT is ~0.194 GHz, which is close to the experimental value of 0.24 GHz, indicating that level repulsion is present even if the equilibrium magnetizations are symmetric, but is due to this much more subtle interaction with the DMI which only manifests in terms of the relative oscillations between the two. When $\mathbf{D} = 0\ or \parallel \hat{\mathbf{y}}$, there is no anticrossing gap indicating a degenerate crossing, but when the DMI is parallel to the applied field, the crossing degeneracy is forbidden, as we have expected. Therefore, we have theoretically determined the coupling strength, and shown that it is dependent on the angle between the applied field and the DMI vector ($\mathbf{H_{ext}} \cdot \mathbf{D}$).

III.2. Analytical LLG model for the passive DMI

Above we have shown that the observed anticrossing gap is well explained by only considering the active contribution of DMI, as opposed to interpreting the DMI as a method to tilt the spins OOP and then using the theory for oblique magnetic fields. Here, we calculate the DMI-induced crossing by only considering a symmetry broken equilibrium magnetic configuration and ignoring the time-dependent contributions in the LLG equations and show that the crossing gap is reduced. The resulting matrix equation for sublattice magnetizations tilted OOP by an angle β has already been determined, we simply apply the result[9].

For these calculations, we will use the same DMI strength as the previous section, as well as the tilting angle β consistent with previous studies. At zero field, the sublattice magnetizations are aligned along the x-axis, only at sufficiently large field does the first order spin flop transition occur to orient them along the y-axis. After this transition, the weak ferromagnetism manifests and the canting angle with respect to the xy plane is $\sin\beta = \frac{D_x}{M_s}\cos\varphi$ where $\beta \sim 4°$. So we can calculate explicitly the anticrossing gap, shown in **Figure S7**. The strength of the magnon-magnon coupling by only considering the presence of the DMI to tilt the spins OOP is ~0.052 GHz, which is much smaller than the measured value. Clearly, then, we have shown that while the DMI does cant the spins OOP and that this OOP tilting can induce the hybridization, we must not neglect the effects of the DMI as it relates to the elliptical oscillations of the magnetization. These oscillations represent a time-dependent magnetic interaction that amplifies the effects of magnon-magnon coupling. This opens the door to begin to search for new magnonic systems with equilibrium magnetizations that are symmetric under twofold rotation symmetry but are still hybridized.

Because β is relatively small, we have shown that the active contribution of the DMI dominates the magnon-magnon coupling phenomenon, instead of the passive contribution. Our fitting results using this model provide excellent results compared to previous measurements on the strength of the DMI in this material.

III.3. Analytical LLG model for the gap at $H = 2H_E$

Here, we present the matrix elements with D_x in the rotated frame as described in section III.2. The rotated frame is tilted OOP by an angle β to produce new unit vectors, $\widehat{x'}$, $\widehat{y'}$, and $\widehat{z'}$

where $\begin{matrix} \widehat{x'} \\ \widehat{y'} \\ \widehat{z'} \end{matrix} = \begin{matrix} \cos(\beta) & 0 & \sin(\beta) \\ 0 & 1 & 0 \\ -\sin(\beta) & 0 & \cos(\beta) \end{matrix} \begin{matrix} \hat{x} \\ \hat{y} \\ \hat{z} \end{matrix}$. The matrix elements in the equation:

$$i\omega \begin{matrix} \boldsymbol{\delta m_+} \\ \boldsymbol{\delta m_-} \end{matrix} = \mu_0\gamma \begin{bmatrix} A & C \\ D & B \end{bmatrix} \begin{matrix} \boldsymbol{\delta m_+} \\ \boldsymbol{\delta m_-} \end{matrix}$$

Become:

$$A = \begin{bmatrix} 0 & -M_s\cos^2\beta \\ 2H_E\cos^2\varphi + M_s\sin^2\beta\cos^2\varphi + D_x\sin\beta\sin 2\varphi & 0 \end{bmatrix}$$

$$B = \begin{bmatrix} 0 & -2H_E - M_s\cos^2\beta \\ 2H_E\sin^2\varphi + M_s\sin^2\beta\cos^2\varphi + D_x\sin\beta\sin 2\varphi & 0 \end{bmatrix}$$

$$C = \begin{bmatrix} -M_s\cos\beta\sin\beta\cos\varphi + D_x\cos\beta\,(\cos\varphi\sin 2\varphi - \sin\varphi\cos 2\varphi) & 0 \\ 0 & M_s\cos\beta\sin\beta\cos\varphi + D_x\cos\beta\sin\varphi \end{bmatrix}$$

$$D = \begin{bmatrix} -M_s\cos\beta\sin\beta\cos\varphi - D_x\cos\beta\,(\cos\varphi\sin 2\varphi - \sin\varphi\cos 2\varphi) & 0 \\ 0 & M_s\cos\beta\sin\beta\cos\varphi - D_x\cos\beta\sin\varphi \end{bmatrix}$$

The key feature here is the frequency of the bare optical mode ($\boldsymbol{\delta m}_+$) at $H = 2H_E$. This frequency is found by subtracting $\begin{bmatrix} i\omega & 0 \\ 0 & i\omega \end{bmatrix}$ and calculating the determinant. When $\varphi = 90°$, the sublattices are exactly aligned and the frequency is identically zero. However, in the rotated frame, $\mathbf{D} = D_x\hat{\mathbf{x}} = D_x(\cos\beta\,\widehat{\mathbf{x}'} - \sin\beta\,\widehat{\mathbf{z}'})$. The nonzero component of the DMI vector along $\widehat{\mathbf{z}'}$, which is orthogonal to the net magnetization direction, causes residual spin canting at $H = 2H_E$ and results in a nonzero frequency of the optical mode. At $\beta = 90°$, however, the optical mode returns to a zero frequency goldstone mode, as its frequency is proportional to $\cos\beta$.

IV. Possible additional sources for the observed anticrossing gap

In addition to the DMI, the magnetic anisotropy landscape along the IP direction may also give additional contributions to the observed magnon-magnon coupling, which we have assumed are small when the field is applied along a principal axis of the material. This anisotropy has been previously shown to enable magnon-magnon coupling by breaking the axial symmetry between the spin up and spin down modes of a compensated ferrimagnet. Likewise, the anisotropy may also break the twofold rotation symmetry about the applied field and enable magnon-magnon coupling between the optical and acoustic modes of an easy plane antiferromagnet. The anisotropy energy is a minimum along the x-axis, the easy axis, and continuously increases as it approaches the y-axis (second easy axis). Therefore, the anisotropy is symmetric under C_2 if the magnetic field is applied along either axis shown in **Figures S8a or S8c**. However, if the magnetic field is applied along an intermediate direction to one of these axes, the twofold rotation symmetry is broken. This will lead to a spin structure that is canted preferentially toward the easy axis and will induce magnon-magnon coupling.

V. Observation of magnon dark modes

The optical mode in the transverse pumping configuration, as well as the optic mode above $|\mathbf{H_{ext}}| = 2H_E$ in any configuration is considered a dark mode, meaning that it cannot be accessed by the spatially uniform microwave field which is applied during our measurement. These selection rules have long since been known and are consistent if one considers the parallel alignment of the sublattices at high field. However, we will show here that the existence of the antisymmetric DMI term will convert the driving microwave field into an effective microwave field which can be used to excite these dark modes.

In **Figure S13a**, the illustration of the magnon 'dark' and 'bright' modes correspond to the optical and acoustic modes under parallel alignment of the sublattices. The DMI Hamiltonian $\mathrm{H}_{DMI} = -\mathbf{D}\cdot(\hat{\mathbf{m}}_\mathbf{A}\times\hat{\mathbf{m}}_\mathbf{B})$ can be rewritten as $\mathrm{H}_{DMI} = \hat{\mathbf{m}}_\mathbf{A}\cdot(D_x\,(\hat{\mathbf{x}}\times\hat{\mathbf{m}}_\mathbf{B})) + \hat{\mathbf{m}}_\mathbf{B}\cdot(-D_x\,(\hat{\mathbf{x}}\times\hat{\mathbf{m}}_\mathbf{A}))$ which exposes the effective fields that each sublattice experiences due to the deflection of the conjugate sublattice. In **Figure S13b**, an OOP rf microwave field generates a torque on both sublattices along the same direction, denoted as $\boldsymbol{\tau}_\mathbf{p}$ for 'primary'. Through these

effective fields in the Hamiltonian, the resulting motion of the sublattices results in a secondary torque which can access the dark mode, denoted as $\boldsymbol{\tau}_{\mathbf{DMI}}$. Therefore, the spatial non-uniformity is realized in these applied torques. Additionally, these effective fields change sign at the same frequency as the driving microwave field, and are even under twofold rotation (C_{2x}). Therefore, our interpretation of these fields in terms of an effective microwave field that can access the optic mode is validated.

VI. Numeric simulation

All features in FMR spectra are in good agreement with the simulation of the precession amplitude, as shown in **Figure S14**. The simulation was run with MATLAB and numerically calculates the time evolution of the magnetization of the two sublattices according to LLG equation (**Eq. S1**), and then the precession amplitude is obtained by fast Fourier transformation. The parameters in the LLG equation are $\mu_0 M_s = 155\ mT$ is the easy-plane anisotropy, Bex = 7.5 mT is the exchange between the two sublattices, $\mu_0 D_x = 11mT$ is the DM vector, $\mu_0 K_x = 7\ mT\ \hat{x}$ is the uniaxial anisotropy, and $\boldsymbol{\tau}$ is the RF field to excite the magnetization dynamics. A Gilbert damping term is also included with alpha=0.01 for the simulation.

Equation S1.

$$\frac{d\hat{m}_A}{dt} = -\mu_0\gamma\hat{m}_A\times(\vec{H}_{ext}-H_E\hat{m}_B-M_s(\hat{m}_A\cdot\hat{z})\hat{z}+K_x(\hat{m}_A\cdot\hat{x})\hat{x}+D_x(\hat{x}\times\hat{m}_B))+\tau_A$$

$$\frac{d\hat{m}_B}{dt} = -\mu_0\gamma\hat{m}_B\times(\vec{H}_{ext}-H_E\hat{m}_A-M_s(\hat{m}_B\cdot\hat{z})\hat{z}+K_x(\hat{m}_B\cdot\hat{x})\hat{x}-D_x(\hat{x}\times\hat{m}_A))+\tau_B$$

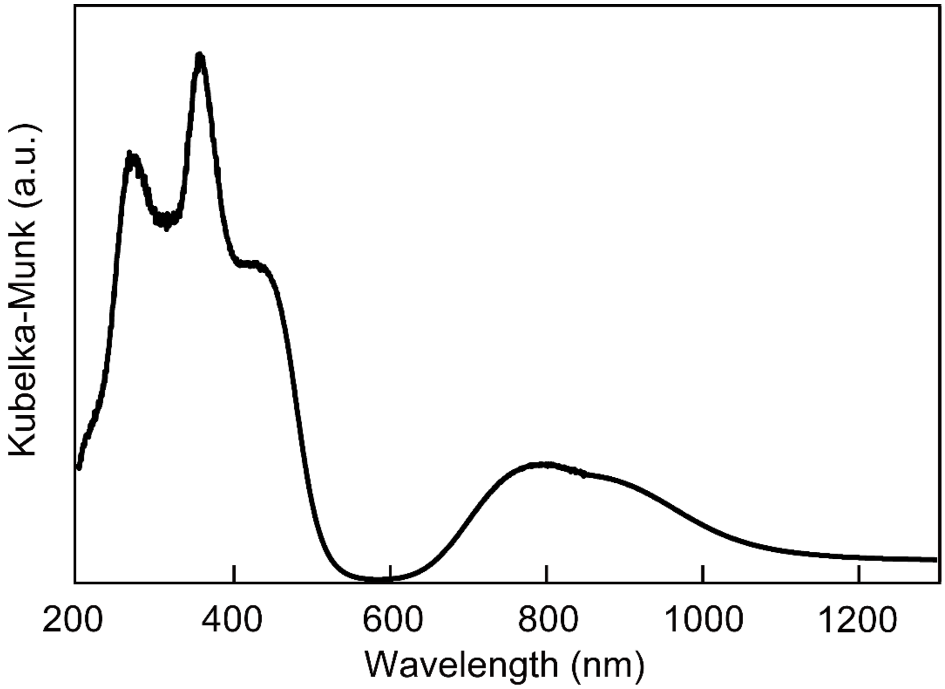

Figure S1.
UV-VIS absorption spectrum of Cu-EA.

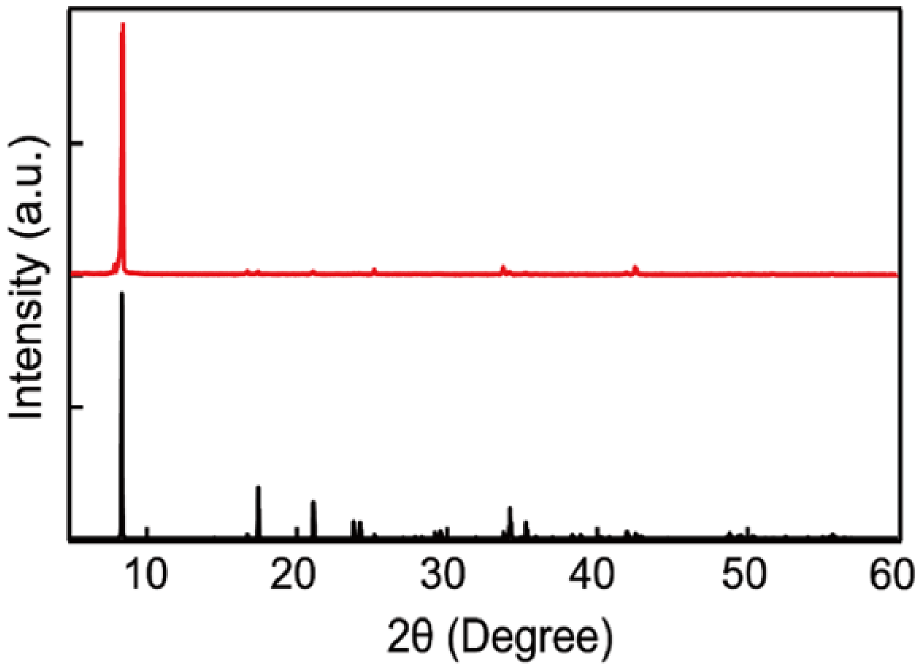

Figure S2.
Powder X-ray diffraction (XRD) data of Cu-EA. The simulated data is based on the reported single crystal data[10].

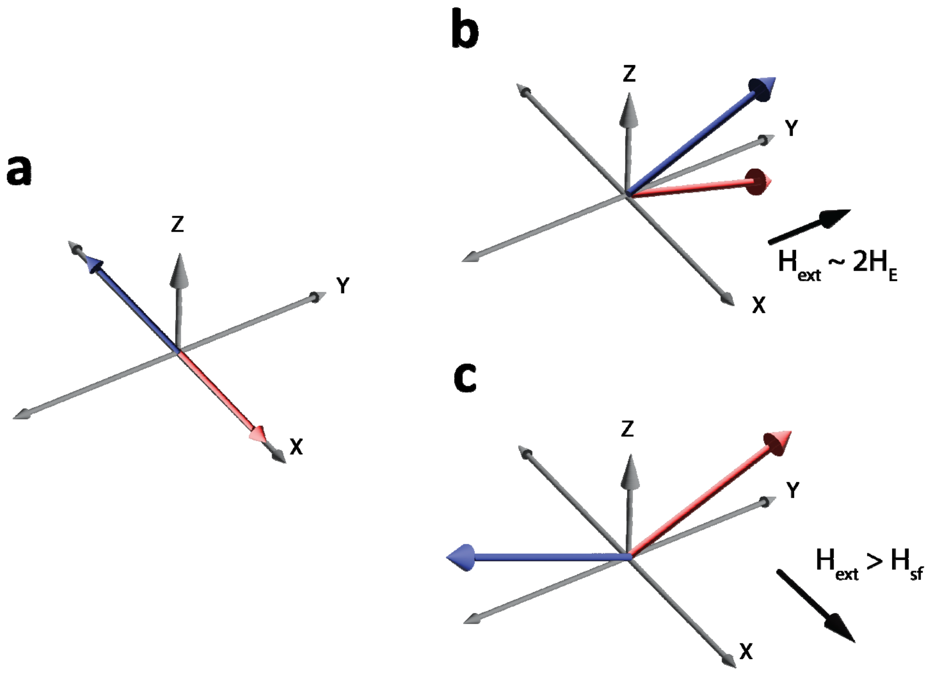

Figure S3.
Spin structure of Cu-EA under different applied fields demonstrating the spin canting which generates a weak ferromagnetic moment along the z-axis in **(c).**

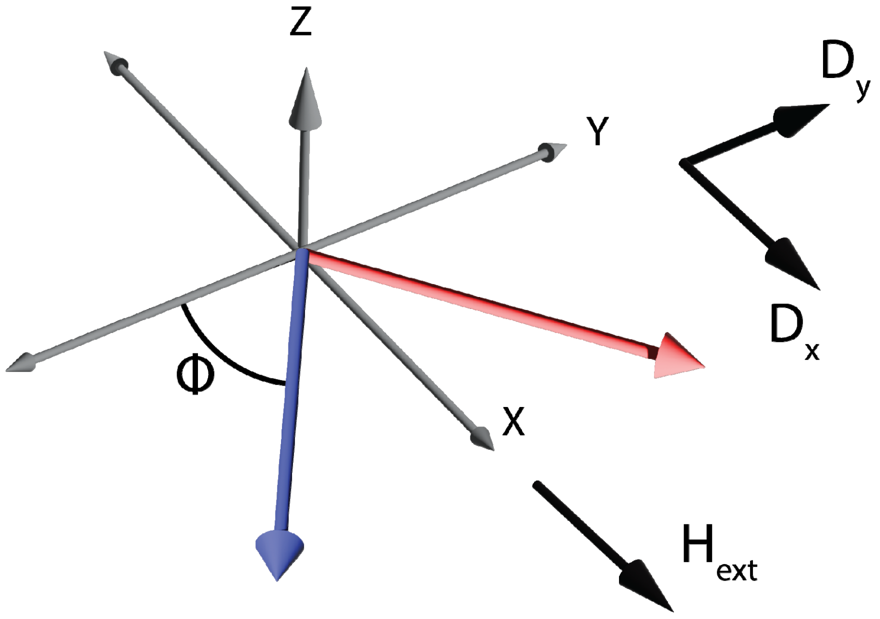

Figure S4.
In-plane alignment of the sublattices by angle φ with the application of the external magnetic field, $\mathbf{H_{ext}}$ to tune the frequencies of the optical and acoustic modes.

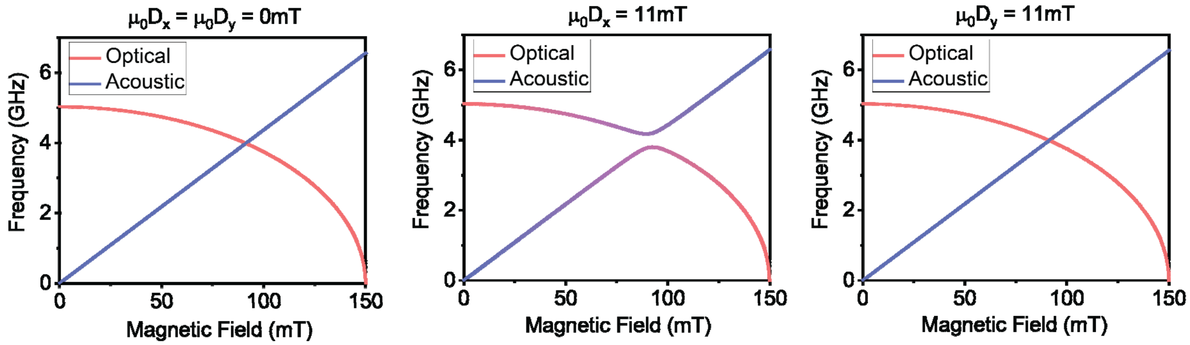

Figure S5.
Calculated solutions for the antiferromagnetic resonance of Cu-EA at different *D* values, ignoring the in-plane magnetic anisotropies and the spin canting contribution.

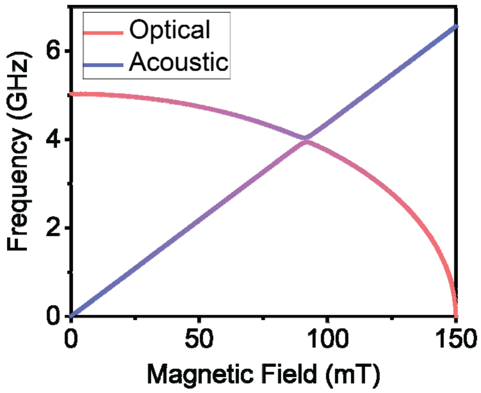

Figure S6.
Magnon mode hybridization due to spin canting only.

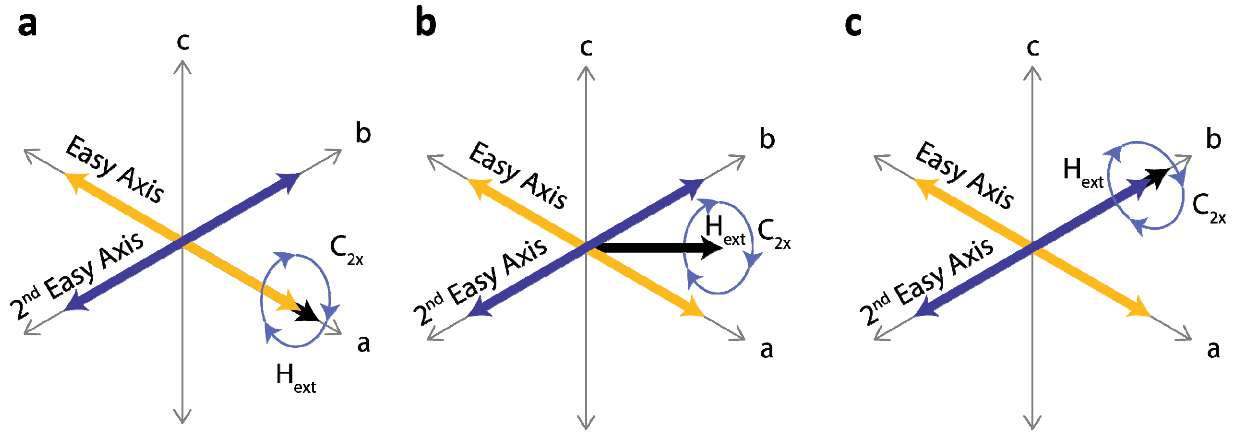

Figure S7.
Interplay of the magnetic anisotropy and symmetry breaking, with symmetric configurations in **a** and **c** and symmetry broken configurations in **b.**

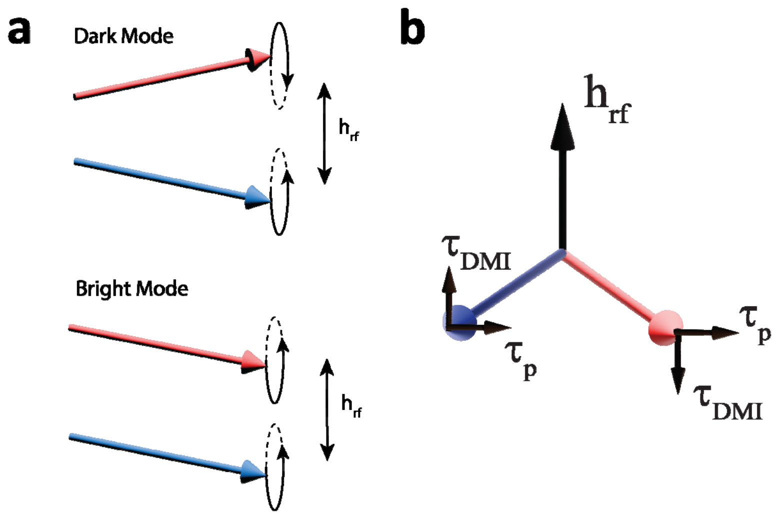

Figure S8.
a, Schematic illustration of so-called ‘dark’ and ‘bright’ modes in the quasi-ferromagnetic phase of the layered antiferromagnet when the sublattices are aligned parallel at $|\mathbf{H}_{\mathbf{ext}}| > 2H_E$. **b,** illustration of the torque from the effective microwave field, h_{rf} which is a product of the motion of the sublattices.

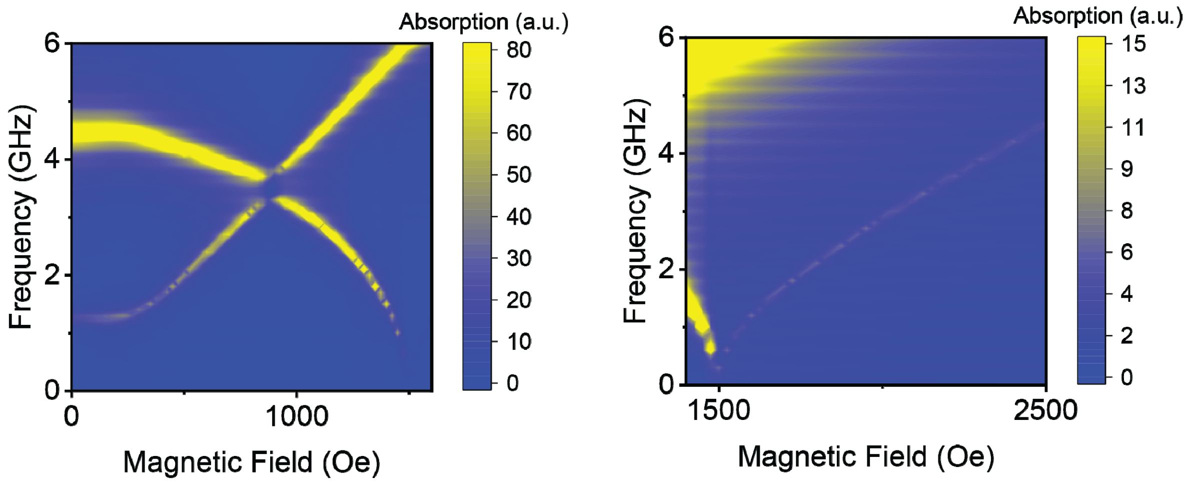

Figure S9.
Numeric LLG simulation showing anticrossing gap and high field optic mode. This simulation was performed with the magnetic field applied at a 45 degree angle to the a-axis to support the data in **Figure 3** of the main text.

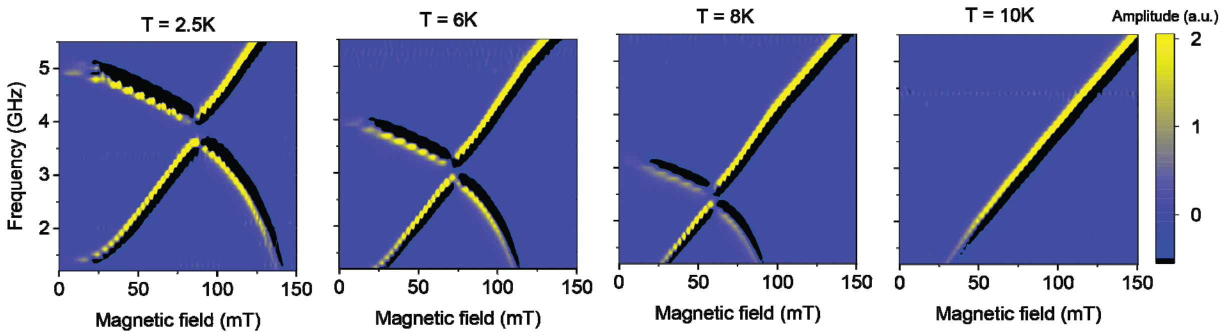

Figure S10.
Temperature dependence of the magnetic resonance, showing a reduction in the optical mode frequency as the temperature approaches 2.5 K, consistent with previously reported results[11]. All measurements are performed in the geometry described in **Figure 3** of the main text.

References

1. Moriya, T. Anisotropic Superexchange Interaction and Weak Ferromagnetism. *Phys. Rev.* **120**, 91–98 (1960).
2. Zolfaghari, P., Wijs, G. A. de & Groot, R. A. de. The electronic structure of organic–inorganic hybrid compounds: (NH4)2CuCl4, (CH3NH3)2CuCl4 and (C2H5NH3)2CuCl4. *J. Phys.: Condens. Matter* **25**, 295502 (2013).
3. Kundys, B. *et al.* Multiferroicity and hydrogen-bond ordering in (C2H5NH3)2CuCl4 featuring dominant ferromagnetic interactions. *Phys. Rev. B* **81**, 224434 (2010).
4. Kimishima, Y. The Magnetic Behaviors of Quasi-Two Dimensional Antiferromagnet (CH3NH3)2CuBr4. *J. Phys. Soc. Jpn.* **49**, 62–66 (1980).
5. Space Group 61: Pbca; P b c a. http://img.chem.ucl.ac.uk/sgp/large/061az1.htm.
6. De Jongh, L. J., Van Amstel, W. D. & Miedema, A. R. Magnetic measurements on (C2H5NH3)2 CuCl4: Ferromagnetic layers coupled by a very weak antiferromagnetic interaction. *Physica* **58**, 277–304 (1972).
7. Liensberger, L. *et al.* Exchange-Enhanced Ultrastrong Magnon-Magnon Coupling in a Compensated Ferrimagnet. *Phys. Rev. Lett.* **123**, 117204 (2019).
8. Bloembergen, P., Berkhout, P. J. & Franse, J. J. M. Static Magnetic Torque Measurements on a System of Ferromagnetic Layers, Coupled by Feeble Antiferromagnetic Interactions; Weak Ferromagnetic Behaviour. *AIP Conference Proceedings* **10**, 1598–1602 (1973).
9. MacNeill, D. *et al.* Gigahertz frequency antiferromagnetic resonance and strong magnon-magnon coupling in the layered crystal CrCl3. *Phys. Rev. Lett.* **123**, 047204 (2019).
10. Steadman, J. P. & Willett, R. D. The crystal structure of (C2H5NH3)2CuCl4. *Inorganica Chimica Acta* **4**, 367–371 (1970).
11. Cham, T. M. J. *et al.* Anisotropic Gigahertz Antiferromagnetic Resonances of the Easy-Axis van der Waals Antiferromagnet CrSBr. *Nano Lett.* **22**, 6716–6723 (2022).
